# Molecular Cytogenetic Analysis of *Deschampsia antarctica* Desv. (*Poaceae*), Maritime Antarctic

**DOI:** 10.1371/journal.pone.0138878

**Published:** 2015-09-22

**Authors:** Alexandra V. Amosova, Nadezhda L. Bolsheva, Tatiana E. Samatadze, Maryana O. Twardovska, Svyatoslav A. Zoshchuk, Igor O. Andreev, Ekaterina D. Badaeva, Viktor A. Kunakh, Olga V. Muravenko

**Affiliations:** 1 Engelhardt Institute of Molecular Biology, Russian Academy of Sciences, Moscow, Russia; 2 Institute of Molecular Biology and Genetics, National Academy of Sciences of Ukraine, Kyiv, Ukraine; Università di Pisa, ITALY

## Abstract

*Deschampsia antarctica* Desv. (*Poaceae*) (2n = 26) is one of the two vascular plants adapted to the harshest environment of the Antarctic. Although the species is a valuable model for study of environmental stress tolerance in plants, its karyotype is still poorly investigated. We firstly conducted a comprehensive molecular cytogenetic analysis of *D*. *antarctica* collected on four islands of the Maritime Antarctic. *D*. *antarctica* karyotypes were studied by Giemsa C- and DAPI/C-banding, Ag-NOR staining, multicolour fluorescence *in situ* hybridization with repeated DNA probes (pTa71, pTa794, telomere repeats, pSc119.2, pAs1) and the GAA simple sequence repeat probe. We also performed sequential rapid *in situ* hybridization with genomic DNA of *D*. *caespitosa*. Two chromosome pairs bearing transcriptionally active 45S rDNA loci and five pairs with 5S rDNA sites were detected. A weak intercalary site of telomere repeats was revealed on the largest chromosome in addition to telomere hybridization signals at terminal positions. This fact confirms indirectly the hypothesis that chromosome fusion might have been the cause of the unusual for cereals chromosome number in this species. Based on patterns of distribution of the examined molecular cytogenetic markers, all chromosomes in karyotypes were identified, and chromosome idiograms of *D*. *antarctica* were constructed. B chromosomes were found in most karyotypes of plants from Darboux Island. A mixoploid plant with mainly triploid cells bearing a Robertsonian rearrangement was detected among typical diploid specimens from Great Jalour Island. The karyotype variability found in *D*. *antarctica* is probably an expression of genome instability induced by environmental stress factors. The differences in C-banding patterns and in chromosome distribution of rDNA loci as well as homologous highly repeated DNA sequences detected between genomes of *D*. *antarctica* and its related species *D*. *caespitosa* indicate that genome reorganization involving coding and noncoding repeated DNA sequences had occurred during the divergence of these species.

## Introduction

The Antarctic Hairgrass, *Deschampsia antarctica* Desv. (*Poaceae*) (2n = 26), is one of the only two flowering plant species found in Antarctica [1, 2]. *D*. *antarctica* has successfully adapted to the harshest environmental conditions (extremely low temperatures, drought, high salinity and flooding, high level of UV radiation, low precipitation). The Antarctic Hairgrass shows tolerance to extreme cold and dry conditions [2–6], ability to become established rapidly [7], and it can photosynthesize at freezing point [8]. The successful Antarctic vegetation of *D*. *antarctica* must have one or various mechanisms which allow the species to live in the extreme environments [3, 5]. One of these mechanisms might be the genomic variability that under environmental stress promotes increasing genome diversity in wild grasses and, consequently, increasing the adaptive potential of populations [9]. The Antarctic Hairgrass could serve as a model for the study of regulation of genome activity as well as for the investigation of the mechanisms responsible for plant adaptation to freezing tolerance [6]. *D*. *antarctica* might also be a useful source of genes associated with stress tolerance and environmental adaptation allowing the development of breeding strategies in agronomical valuable crops [10–11]. Besides, extracts from *D*. *antarctica* are known to display protective effects against ultraviolet radiation used for pharmaceutical purposes [12].

The evident importance of *D*. *antarctica* genome as a unique recourse for such studies has generated a number of research works mainly performed at the morphological, physiological, biochemical or molecular levels [13–14]. In particular, the chloroplast genome has been sequenced and plastid transcriptome profiles of the coding/noncoding genes have been investigated [11, 15]. The Lip3F9 polypeptide with lipase activity from *D*. *antarctica* has been isolated and characterized [16]. A multi-gene family from *D*. *antarctica* encoding ice recrystallization inhibition proteins whose transcript levels are responsive to cold acclimation, has also been characterized [5–6]. However, little nuclear genomic information is still available for this species [6, 15–16].

The results of previous molecular genetic studies with the use of RAPD- and AFLP-analyses showed different levels of genetic diversity in *D*. *antarctica* populations from various regions of Maritime Antarctic [14, 17–21]. It was also shown that environmental stress factors can lead to physiological stress followed by structural variations in plant genomes including chromosome rearrangements, mixoploidy and aneuploidy [14, 22–25]., The comparative molecular cytogenetic analysis of populations of *D*. *antarctica* growing in different areas of the Maritime Antarctic has not been studied yet as the karyotype of *D*. *antarctica* is still poorly investigated. Only the chromosome number 2n = 26 = 2(5m+3sm+4st+1t) with one pair of satellite (SAT) chromosomes had been determined by simple monochrome staining [1, 26]. A detailed knowledge of karyotype structure of *D*. *antarctica* is needed for further chromosome mapping of genes associated with stress tolerance as well as comparative cytogenetic studies within the genus *Deschampsia* for a better understanding of organization, structure, and evolution of this genus.

In the present paper, a comprehensive molecular cytogenetic analysis of *D*. *antarctica* specimens from four islands of the west coast of the Antarctic Peninsula was carried out for the first time. In karyotypes of *D*. *antarctica*, Giemsa C-, DAPI/C-banding patterns and AgNOR staining were studied. Chromosome distribution of 45S and 5S rDNA, telomere repeats, satellite DNA sequences of cereals pSc119.2 and pAs1, the (GAA)n microsatellite sequence as well as homologous highly repeated sequences of genomic DNA of the related species *D*. *caespitosa* were investigated by multicolour fluorescence *in situ* hybridization (FISH) and sequential rapid genomic *in situ* hybridization (rapid GISH).

## Materials and Methods

### Ethics Statement

This study including sample collection and experimental research conducted on these materials was according to the law on activities and environmental protection to Antarctic approved by the Ministry of Education and Science of Ukraine.

### Plant material

The seeds of *D*. *antarctica* growing under natural conditions were collected in the vicinity of the Ukrainian Antarctic Station “Academician Vernadsky”: Galindez Island (northwestern corner of the island at Caroline point: 65°14.955´ S, 64°15.181´ W), Skua Island (northwestern corner of the island: 65°15.302´ S, 64°16.493´ W), Great Jalour Island (northeastern part of the island: 65°14.039´S, 64°9.761´W) and Darboux Island (northern end of the island in the vicinity of a rocky grotto: 65°23.707´ S, 64°12.905´ W). The habitats of *D*. *antarctica* were small (up to 1 km^2^) rocky islands partly covered with snow. The plant material was obtained from the research Antarctic expeditions (seasons 2005–2008) organized by the National Antarctic Scientific Center of Ukraine. The accessions of *D*. *caespitosa* were collected (natural population, Kyiv region, Ukraine) and identified by Dr. I.Yu. Parnikoza, Institute of Molecular Biology and Genetics of National Academy of Sciences of Ukraine (Kyiv, Ukraine). The identification of the species was performed according to the Manual of vascular plants of Ukraine [27].

For obtaining aseptic roots of *D*. *antarctica*, dry seeds were sterilized and germinated as described earlier [28]. The obtained aseptic plants were cultivated on B5 agar nutrient medium [29] supplemented with 0.1 mg/L α-naphthaleneacetic acid (NAA). To produce plant material in sufficient quantity, the plants were *in vitro* propagated through fragmentation of the obtained root mat.

### Fixation

For cell cycle synchronization and accumulation of mitotic divisions, root tips were incubated in ice water for 24 hours at 0°C and then fixed in the ethanol:glacial acetic acid fixative (3:1) for 48 h at room temperature. Fixed roots were stored in the fixative at -20°C before use.

### Chromosome spread preparation

For C-banding, chromosomes spreads were prepared as described previously [30].

For *in situ* hybridization and DAPI staining, before chromosome spread preparation, the roots were stained in 1% acetocarmine solution in 45% acetic acid for 40 min, the tip caps with root meristem were cut on the object-plate, the meristem was macerated in a drop of 45% acetic acid, and then squashed chromosome preparations were made. The cover slips were removed after freezing, and the preparations were dehydrated and stored in 96% ethanol at -20°C.

### C-banding

The C-banding procedure was carried out according to the technique described previously [31].

### Fluorescence *in situ* hybridization

Following probes were used for FISH:

1. pTa71 containing a 9 kb long repeated DNA sequence of common wheat encoding 18S, 5.8S and 26S rRNA genes including spacers [32];
2. pTa794 containing a 420 bp long repeated DNA sequence of wheat containing the 5S rRNA gene and the intergenic spacer [33];
3. pAs1 containing a 1 kb repeated DNA sequence isolated from *Aegilops tauschii* [34];
4. pSc119.2 containing a120 bp long repeated DNA sequence isolated from rye [35];
5. the *Arabidopsis*-type telomere repeat probe (TTTAGGG)_n_ generated by PCR according to [36];
6. the (GAA)_n_-oligonucleotide probe labelled at the 3’-end with fluorescein-12-dUTP (Roche Diagnostics, Mannheim, Germany) was synthesized using a synthesizer ABI 394 (Applied BioSystems, Redwood City, USA).

Dual and multicolour FISH assays were performed using combinations of DNA probes labelled directly with different fluorochromes including SpectrumAqua, SpectrumGreen, SpectrumGold, SpectrumRed (Abbott Molecular, Wiesbaden, Germany) and also labelled indirectly with biotin-16-dUTP (Roche Diagnostics, Mannheim, Germany) or digoxigenin-11-dUTP (Roche Diagnostics, Mannheim, Germany) by nick translation according to manufacturers’ protocols. FISH procedure was conducted as described previously [37].

### DAPI staining

After FISH, chromosome slides were stained with 0.1 μg/ml DAPI (4',6-diamidino-2-phenylindole (DAPI, (Serva, Heidelberg, Germany)) in Vectashield mounting medium (Vector laboratories, Peterborough, UK).

### Rapid GISH procedure

Genomic DNA of *D*. *caespitosa* was isolated from young leaves using CTAB (cetyltrimethylammonium bromide) standard protocol [38] and labelled with SpectrumAqua (Abbott Molecular, Wiesbaden, Germany) by nick translation according to the manufacturer’s instructions. After FISH procedures and documentation of the hybridization patterns, the chromosome slides were washed for 5 minutes in distilled water, dehydrated through the ethanol series, denatured in 70% formamide in 2 × SSC at 73°C for 3 min, dehydrated through the cold ethanol series and air dried. The labelled genomic DNA probe (80 ng) was dissolved in hybridization mixture (10% w/v dextran sulfate, 50% v/v formamide, 1% v/v Tween and 2 × SSC) (with the total volume 15 μL), denatured at 85°C for 5 min, chilled on ice and placed onto the slide. After the hybridization for 40 minutes at 37°C in a moist chamber, the slides were washed and DAPI stained as described previously [37].

### Ag-NOR staining

Ag-NOR staining was performed according to Howell and Black [39]. After Ag-NOR staining slides were mounted in 0.1 μg/ml of DAPI (4',6-diamidino-2-phenylindole) (Serva, Heidelberg, Germany) in Vectashield medium (Vector laboratories, Peterborough, UK) for chromosome visualization.

### Chromosome analysis

The slides were examined using Olympus BX61 epifluorescence microscope (Olympus, Tokyo, Japan). Images were taken with monochrome CCD camera (Cool Snap, Roper Scientific Inc., Tucson, USA) and sequentially collected in grayscale channels. Then, they were pseudocoloured and processed with Adobe Photoshop 10.0 (Adobe Systems Inc., USA) software. At least five specimens of *D*. *antarctica* from each island and fifteen metaphase plates from each specimen were analyzed.

The chromosome images were measured using an image analysis system “Videotest-Karyo 1.5” (IstaVideotest, St Petersburg, Russia). Based on the results of measurements of 10 metaphase plates from 5 individual plants, relative chromosome length (100 x chromosome length/total haploid length) and centromeric index (100 x length of short arm/total chromosome length) were calculated. The chromosome pairs were classified according to their centromeric position [40] in the decreasing order of size.

## Results

### C- and DAPI/C-banding analysis

**Fig 1:**
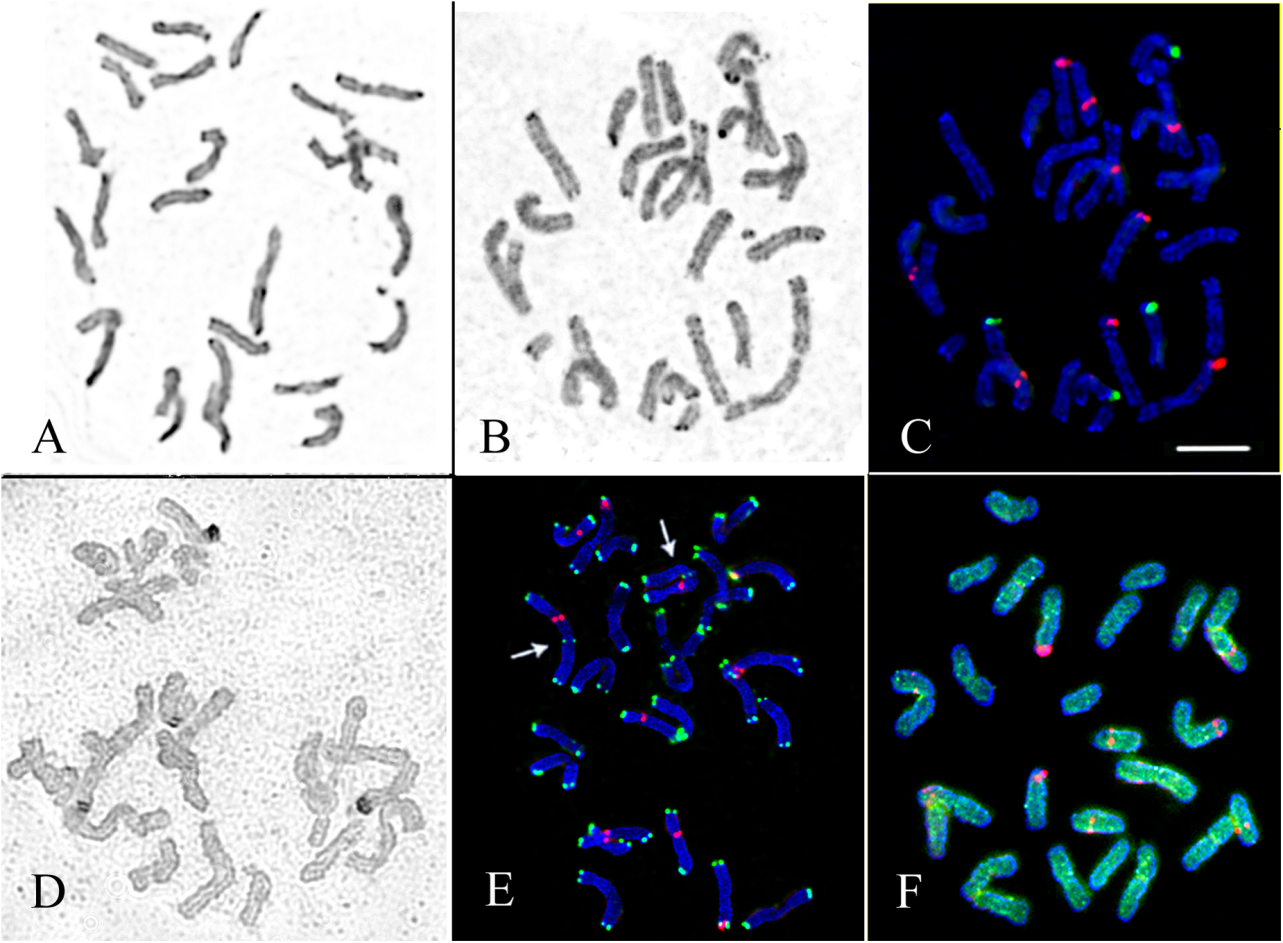
Chromosome spreads of *D*. *antarctica*. (A) Giemsa C-banded chromosomes of the specimen from Galindez Island. (B) Inverted image of the DAPI/C-banded karyotype and (C) localization of 45S (green) and 5S (red) rDNA sites on chromosomes of the specimen from Galindez Island. (D) Ag-NOR staining patterns (dark segments) of chromosomes of the specimen from Skua Island. (E) Localization of telomeric repeats (green), 45S (green) and 5S (red) rDNA loci and in the karyotype of the specimen from Skua Island. Arrows point to the intercalary loci of telomere repeats detected on the largest chromosome pair. (F) Distribution of 5S rDNA sites (red) and GAA microsatellite sequence (green) on chromosomes of the specimen from Skua Island. Scale bar—5 μm.

**Fig 2:**
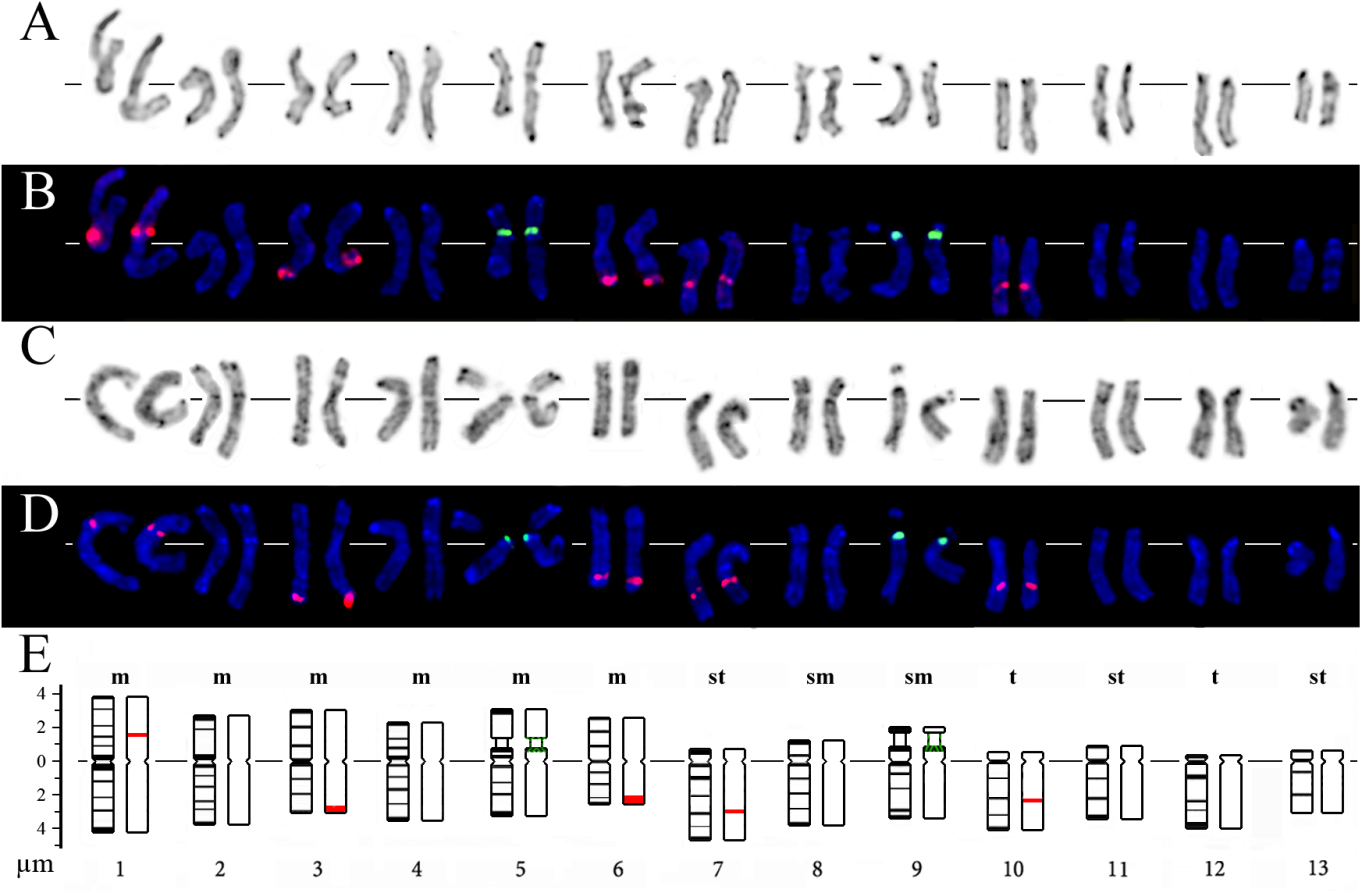
Karyograms and idiograms of *D*. *antarctica* chromosomes. Karyograms after (A) Giemsa C-banding and (B) FISH with 45S (green) and 5S (red) rDNA probes (the same metaphase plate as in Fig 1A). Karyograms after (C) DAPI/C-banding (inverted image) and (D) FISH with 45S (green) and 5S (red) rDNA (the same metaphase plate as in Fig 1B and 1C). (E) Idiograms of *D*. *antarctica* showing relative sizes and positions of DAPI/C-bands (black segments), 45S (green) and 5S rDNA (red).

*D*. *antarctica* specimens with typical diploid karyotypes (2n = 26) were found on all four islands (Galindez, Darboux, Skua and Great Jalour) of the Maritime Antarctic (Figs 1 and 2). Chromosomes in the karyotypes were subdivided into four groups based on their centromeric position and morphology. The first group comprised six metacentric chromosomes, the second group–two submetacentric chromosomes, the third group–three subtelocentric, and the fourth group–two telocentric chromosomes. Accordingly, the karyotype formula was 2n = 26 = 2(6m+2sm+3st+2t).

**Fig 3:**
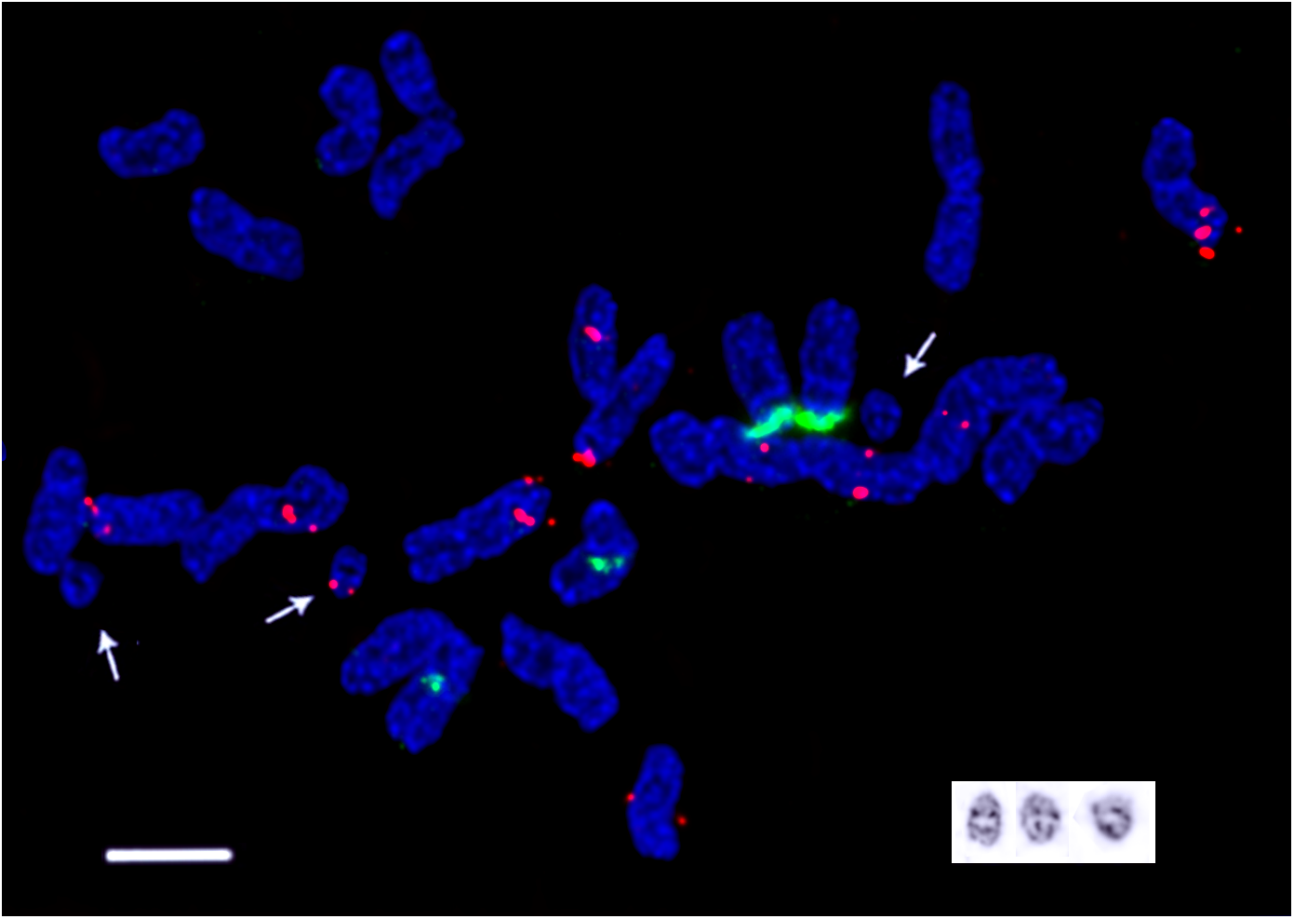
Chromosome spread of *D*. *antarctica* specimen from Darboux Island. Chromosome localization of 45S (green) and 5S (red) rDNA sites and inverted image of DAPI/C-banded B-chromosomes (bottom right). Arrows point to the B-chromosomes. Scale bar—5 μm.

In karyotypes of most of the specimens from Darboux Island, 1–3 supernumerary chromosomes (B chromosomes) were observed (Fig 3). Also, a mixoploid plant was detected among the specimens from Great Jalour Island. The analysis of 82 metaphases from six roots of the plant showed that the chromosome number ranged from 27 to 54, and 67 metaphases had 38 chromosomes (modal number).

C-banding analysis of *D*. *antarctica* showed similar distribution of Giemsa C-bands in karyotypes of the specimens from different islands: large C-bands were located in the pericentromeric and telomeric regions of chromosomes, while a number of small and also faint and inconsistent C-bands were detected in the interstitial chromosome regions (Figs 1A and 2A).

After FISH procedures, chromosome staining with DAPI revealed C-like banding patterns (DAPI/C-banding patterns) (Figs 1B and 2C) which became more evident after several sequential FISH procedures.

Visual analysis of Giemsa C- and DAPI/C-banding patterns of the specimens indicated small variations in size and number of the intercalary and telomeric bands as well as C-bands adjacent to the secondary constriction regions of the satellite chromosomes. Bs, found in karyotypes of the specimens from Darboux Island, possessed distinct heterochromatic bands in their telomeric regions (Fig 3).

### Chromosome localization of 45S and 5S rDNA sites

FISH-analysis showed that 45S rDNA loci were localized on two chromosome pairs: in the proximal region of the short arm of a metacentric chromosome pair and in the distal region of the short arm of a submetacentric chromosome pair. The secondary constriction of the submetacentric chromosome was well-defined, and the 45S rDNA site was larger (though the satellite was smaller) compared with the other SAT chromosome pair (Figs 1A, 1B, 1C, 2 and 3). We observed 5S rDNA sites on five chromosome pairs: in the proximal region of the short arm of the largest metacentric chromosome, in the subtelomeric regions of the long arms of two other metacentric chromosomes, in the proximal regions of the long arms of a pair of subtelocentric chromosomes and a pair of telocentric chromosomes. 5S rDNA loci were smaller and more polymorphic in size than 45S rDNA loci (Figs 1C, 1E, 1F, 2B and 2D). Besides, weak 5S rDNA sites were detected in the subtelomeric regions of one of the Bs found in some kariotypes of *D*. *antarctica* from Darboux Island (Fig 3).

### Detection of active nucleolus organizer regions by Ag-NOR staining

In diploid metaphase spreads, Ag-NOR staining revealed four stained regions in two chromosome pairs indicating that the NORs were transcriptionally active in both SAT chromosomes. Two Ag-NORs are clearly more intense than the other two (Fig 1D).

### Chromosome localization of telomere repeats

**Fig 4:**
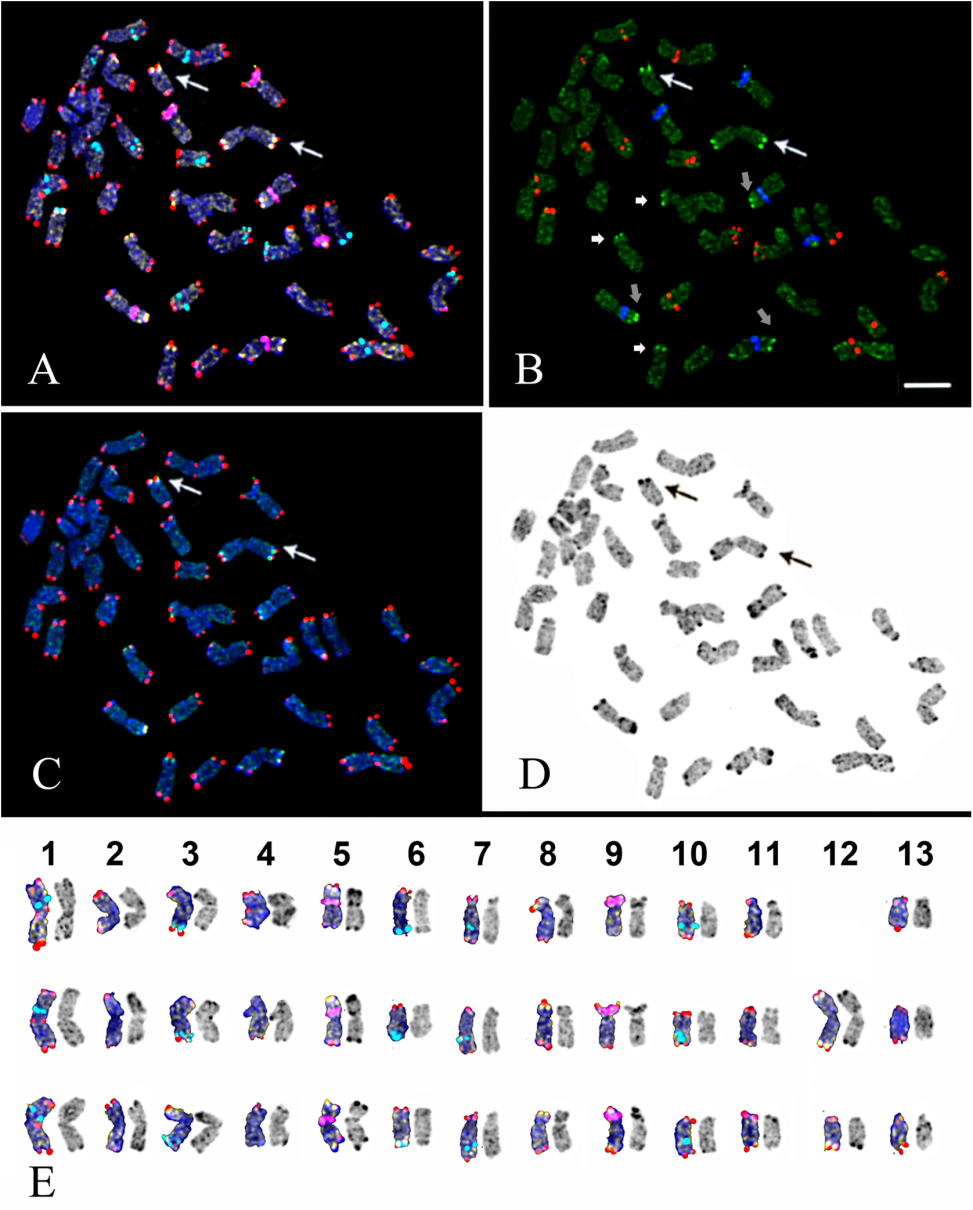
Chromosome spread of the triploid *D*. *antarctica* specimen from Great Jalour Island. (A) The merged fluorescent image: multicolour FISH with telomeric repeats (red), 45S (purple) and 5S (light blue) rDNA probes and sequential rapid GISH with genomic DNA of *D*. *caespitoza* (yellow). (B) Localization of chromosome markers revealed by rapid GISH (light green) in relation to 45S (blue) and 5S (red) rDNA loci. Arrows indicate the brightest green signals detected on chromosomes 5 (grey arrows), 8 (short arrows) and 12 (long arrows). (C) Chromosome markers revealed by rapid GISH (yellow) in relation to telomeric repeat signals (red). (D) Inverted image of DAPI/C-banded chromosomes. Long arrows indicate chromosome 12 and a Robertsonian translocation between two homologous chromosomes 12. (E) Karyograms of metaphase chromosomes after multicolour FISH (left) and DAPI/C-banding (inverted image) (right). Scale bar—5 μm.

In FISH assays, the sites of telomere repeat sequences were detected in the terminal regions of all chromosomes. Additionally, in karyotypes of all the specimens, a polymorphic interstitial hybridization signal of the telomere repeats was localized in the proximal position of the long arm of the largest chromosome (Figs 1E, 4A and 4C).

### FISH mapping of pAs1, pSc119.2 and GAA-oligonucleotide probes

FISH analysis revealed dispersed hybridization sites of the GAA microsatellite sequence along the chromosomes of *D*. *antarctica* (Fig 1F). Hybridization signals of pAs1 or pSc119.2 probes were not visualized.

### Chromosome identification and karyotype analysis

Based on chromosome morphology, Giemsa C- and DAPI/C-banding patterns and also results of FISH analysis, all chromosomes in the karyotypes of *D*. *antarctica* were identified. Chromosome idiograms of typical diploid *D*. *antarctica* including Giemsa C- and DAPI/C-banding patterns as well as 45S and 5S rDNA localization were constructed (Fig 2E).

The analysis of DAPI/C-banding patterns of the 38-chromosome karyotype of mixoplod *D*. *antarctica* from Great Jalour Island allowed us to conclude that it was a triploid possessing a chromosome rearrangement (presumably chromosome fusion) (Fig 4D and 4E). FISH analysis showed that the rearranged chromosome did not possess any chromosome markers. Accordingly, we performed a rapid GISH procedure using labelled genomic DNA of the related species *D*. *caespitosa* as a probe. The hybridization procedure revealed bright signals in the subtelomeric regions of the short arms of chromosomes 5, 8 and 12. Two of the homologous telocentric chromosomes 12 were involved in the Robertsonian rearrangement **(**centric fusion translocation) (Fig 4 arrows).

The Robertsonian translocation t(12; 12) was observed in most of the cells of the mixoploid plant. Two tetraploid metaphases comprised four haploid chromosome sets (4n = 52) without translocations. Also, two chromosome translocations t(12; 12) together with four haploid chromosome sets were found in one metaphase having 54 chromosomes. The number of chromosomes in the other metaphase plates varied from 27 to 48, but it was not possible to determine whether or not the cell was aneuploid as some of the chromosomes could be lost during slide preparation (Table 1).

**Table 1:** Cytogenetic analysis of *D*. *antarctica* specimens from Great Jalour Island.

Number of plants	Genotype	Number of roots	Number of chromosomes	Number of metaphases	Number of t(12; 12)	Ploidy level
4	Typical diploid	7	26	67	0	2x = 26
1	Mixoploid	1	27	1	1	2x = 26+t(12; 12)
			36	1	1	
			38	9	1	3x = 37+t(12; 12)
			48	1	1	
			54	1	2	4x = 52+2t(12; 12)
		1	32	1	1	
			35	1	0	
			36	1	1	
			38	10	1	3x = 37+t(12; 12)
			45	1	1	
			52	2	0	4x = 52
		1	33	1	1	
			38	13	1	3x = 37+t(12; 12)
		1	34	1	1	
			36	1	1	
			38	12	1	3x = 37+t(12; 12)
		1	37	1	1	
			38	11	1	3x = 37+t(12; 12)
		1	36	1	1	
			38	12	1	3x = 37+t(12; 12)

## Discussion

*Deschampsia* P. Beauv. is a cosmopolitan genus that involves a group of taxa with a wide geographical distribution [26, 41]. The high morphological diversity makes the taxonomy of the genus very difficult [26]. Most of *Deschampsia* species have an unusual for cereals basic chromosome number x = 13 with the exception of *D*. *setacea* (2n = 2x = 14), *D*. *atropurpurea* (2n = 2x = 14) and *D*. *fluxuosa* (2n = 4x = 28) which present a basic number of x = 7 [26]. A molecular phylogenetic study of *Deschampsia* based on the analysis of nuclear ribosomal internal transcribed spacer (ITS) and plastid *trnL* intron sequences showed that *D*. *antropurpurea* and *D*. *fluxuosa* should be excluded from the genus *Deschampsia*, while the core of the genus *Deschampsia* was mainly represented by the subspecies of *D*. *caespitosa* (*D*. *caespitosa* complex) [42]. The *D*. *caespitosa* complex is the most studied taxon in the genus with a widespread distribution including extreme polar environments [41, 42]. In *D*. *caespitosa*, different karyotypes were described [41–43], and also inter- and intraindividual variability in chromosome number (2n = 15–28) as well as different ploidy levels (2x = 26; 3x = 39; 4x = 52) were reported [41–44].

*D*. *antarctica*, according to the phylogenetic studies based on the analysis of nuclear ribosomal ITS and plastid sequences, was included in the core of the genus *Deschampsia* [45, 46]. However, in order to clarify the phylogenetic relationships of this species within the genus, the need for obtaining data of different geographical origins and ploidy levels has been pointed out [26, 46].

In the present study, a cytogenetic analysis of *D*. *antarctica* specimens from four localities of Maritime Antarctic also revealed 13 chromosome pairs in typical diploid karyotypes. However, morphological analysis of chromosomes detected minor differences in the karyotype formula (2n = 26 = 2(6m+2sm+3st+2t)) compared to the earlier described one (2n = 26 = 2(5m+3sm+4st+t)) [26]. This is probably due to the fact that we have used chromosome specific markers for more precise identification of homologous pairs.

Apart from the chromosome number, similar features in the karyotype structure of *D*. *antarctica* and *D*. *caespitosa* were revealed [26, 41], in particular, their karyotypes contain six pairs of metacentric chromosomes with one pair being almost twice as long as the others and seven pairs of acrocentric chromosomes [26, 42]. The appearance of such a long metacentric chromosome in karyotypes of these species could be a result of a translocation (or a centric fusion) between two normal sized acrocentric chromosomes, giving rise to the unusual for cereals chromosome number 2n = 26 [41]. The additional intercalary signal of telomere repeats detected in the largest chromosome 1 indirectly confirms this assumption because intercalary sites of telomere repeats have been considered to be the remnants of ancestral chromosome fusions (or other rearrangements) produced during the evolution of karyotypes [47].

In karyotypes of the most of *D*. *antarctica* specimens from Darboux Island, we found supernumerary chromosomes (B chromosomes). Due to the dispensable nature of Bs, they can be present or absent among individuals of the same population in a species [48–49]. It should be noticed that B chromosomes were previously detected in karyotypes of other species of the D. *caespitosa* complex [14]. Also, the correlation between the appearance of Bs in a karyotype and environmental conditions was found [48, 50–51]. Interestingly, the Bs were found only in karyotypes of *D*. *antarctica* from the southernmost Darboux Island (correspondingly, exposed to the harshest environmental conditions). Nevertheless, there is a strong possibility that Bs can exist in karyotypes of *D*. *antarctica* established on the other islands as not all the clusters (populations) of plants were studied.

Furthermore, apart from typical diploid specimens of *D*. *antarctica*, a mixoploid plant with mainly triploid cells bearing the Robertsonian rearrangement between homologous chromosomes was found among the samples from Great Jalour Island. In harsh conditions of Maritime Antarctic, where the areas available for colonization by the species are restricted to small isolated clusters among rocks and rock cracks, one of the types of propagation (sexual or asexual) could gain an advantage over the other one. The chromosome rearrangement could appear in the population under the influence of some stress factors. The meiotic mutations and/or apomictic propagation had maintained it as a triploid seed, and after the seed germination, mixoploidy was detected in the roots. Our observations are in agreement with early reported suggestions that the presence of mixoploid plants in *D*. *antarctica* populations is maintained by its capacity of vegetative and/or apomictic propagation [14; 52]. It has been shown that apomixis is accompanied with phenomena of poly-, aneu-, and mixoploidy in flowering plants [53, 54]. Also, a high level of aneuploidy has been found in plant species propagated asexually [55].

The karyotype variability revealed in the present study might be a manifestation of the cytogenetic abnormalities (mixoploidy, polyploidy, chromosome rearrangements and appearance of B chromosomes) which are especially common in plants growing under various environmental stresses [22, 24–25]. Environmental stress-induced inter and intra individual variability in chromosome number, aneuploidy and aneusomaty were early found in *D*. *caespitosa* [42, 44, 46]. Interestingly, in *D*. *antarctica*, which is the only *Deschampsia* species adapted to the harsh environment of the Antarctic, we observed almost the full range of karyotype variability found in the whole genus.

Although monochrome staining detected evident karyotype similarities in *D*. *antarctica* and *D*. *caespitosa*, detailed molecular cytogenetic analysis revealed structural differences between them. Particularly, Giemsa C-banding analysis showed a smaller amount of C heterochromatin in karyotypes of *D*. *antarctica* specimens compared to *D*. *caespitosa* [41]. The observed C-banding patterns in *D*. *antarctica* karyotype were more like the C-banding patterns of *D*. *setacea* (2n = 14) and *D*. *fluxuosa* (2n = 28) [41] as well as some diploid *Avena* L. species (2n = 14) [56] than in *D*. *caespitosa* (2n = 26) which mostly had large telomeric C-positive bands in the long arms of the chromosomes but lacked diffuse heterochromatin [41]. Although we found very few published studies on Giemsa C-banding analysis within the genus *Deschampsia* [41], and further investigation is needed, these observations indicate that genome reorganization involving repeated DNA sequences had occurred during the divergence of *D*. *antarctica* and its related species *D*. *caespitosa*.

Analysis of C-banding patterns in karyotypes of the examined specimens of *D*. *antarctica* from different islands revealed a low level of polymorphism between the studied populations in the number and size of intercalary, pericentromeric and telomeric C-bands as well as the bands adjacent to the secondary constriction regions of the SAT chromosomes. This may be due to the narrow distribution area of the species. Nevertheless, further detailed investigation of the intraspecific variation in C-banding patterns is needed in order to investigate the possible influence of the harsh environments of Antarctica's interior on C-heterochromatin content in this extremophile.

After FISH, that includes denaturation and renaturation of chromosome DNA, DAPI staining displays AT-rich heterochromatin stains as DAPI(+) bands and reveals Giemsa C-banding-like patterns (DAPI/C-banding patterns) in karyotypes of vascular plants [57]. DAPI/C-banding, together with localization of rDNA loci, provides a useful tool to detect chromosome variations among populations and closely related species [58–59]. Such approach allowed us to identify all the chromosome pairs in *D*. *antarctica* karyotypes and construct chromosome idiograms of the species using Giemsa C- and DAPI/C-banding patterns as well as 45S and 5S rDNA localization. The obtained results are necessary for karyotype comparisons and clarifying phylogenetic relationships within the genus *Deschampsia*.

FISH analysis revealed differences in chromosome localization of rDNA loci between karyotypes of *D*. *antarctica* and *D*. *caespitosa*. In *D*. *antarctica*, we detected 45S rDNA sites in two chromosome pairs. Only one of the SAT chromosome pairs possessed a well-defined secondary constriction, and this was probably the reason why monochrome staining had previously revealed only one SAT chromosome pair in *D*. *antarctica* karyotype [26]. In comparison, the karyotype of *D*. *caespitosa* was shown to comprise three SAT chromosome pairs bearing 45S rDNA [43]. The absence of one pair of SAT chromosomes with proximal 45S rDNA in *D*. *antarctica* karyotype could be due to chromosome rearrangements occurred during the speciation process. Using Ag-NOR staining specific to transcriptionally active NORs [60–61], we found that the NORs of both SAT chromosomes in *D*. *antarctica* were functionally active. However, the transcriptional activity of the NORs in *D*. *caespitosa* as well as in other *Deschampsia* species has not been studied yet, and further investigation of the NORs origin is required.

The number and chromosome localization of 5S rDNA sites in *D*. *antarctica* karyotype found in the present study, differed from the distribution of 5S rDNA in *D*. *caespitosa* described earlier. In *D*. *caespitosa* karyotype, 5S rDNA were localized in four chromosome pairs, one of which had 5S rDNA bands in both arms [43]. In *D*. *antarctica*, ten 5S rDNA loci were found on five chromosome pairs. Besides, 5S rDNA site was detected in one of the Bs found in karyotypes of *D*. *antarctica* specimens from Darboux Island. It was previously shown that B chromosomes detected in karyotypes of some *Poaceae* species contained ribosomal genes as well as pSc119.2 tandem repeats [24, 62–65]. In agreement with these observations, our results showed that in karyotypes of *D*. *antarctica* specimens from Darboux Island there were at least two types of B chromosomes which possessed distinct DAPI/C-positive bands and one of them contained 5S rDNA sequences.

Thus, the revealed differences in C-heterochromatin content and in numbers and distribution of rDNA sites between genomes of *D*. *antarctica* and *D*. *caespitosa* showed that genome reorganization involving coding and noncoding repeated DNA sequences occurred during the divergence of the species.

Vascular plants are known to be enriched with repetitive DNAs which may constitute up to 95% (in *Allium cepa*) of the total genomic DNA and play an important role in the speciation processes [66–68]. Knowledge of the distribution, genomic organization and chromosome localization of highly repeated DNA sequences in plants as well as in their ancestors provides information about their reorganization during evolution [69]. It should be noticed that phylogenetic position of the genus *Deschampsia* within the family *Poaceae* is still controversial. Based on classical morphological traits, the genus was considered to belong to the *Avenea* tribe [41, 70]. However, recent molecular genetic studies have shown alternative phylogenetic positions of *Deschampsia* (i.e., *Aveneae* or *Poeae* tribes) depending on the target sequences used for examination or the parameters used for grouping [15, 45, 71–73]. Accordingly, we performed FISH analysis of *D*. *antarctica* using several DNA probes specific for members of the family *Poaceae* (that includes both *Aveneae* and *Poeae* tribes).

Recently, microsatellite DNA sequences have become one of the most widely used molecular markers for genetic studies as they are major components of many plant genomes. The GAA-microsatellite sequence was found to distribute in genomes of different cereals [74], and FISH with the GAA microsatellite sequence as a probe produced banding patterns similar to those obtained by N-banding in chromosomes of some cereals [75]. In the present study, FISH analysis revealed dispersed hybridization sites of the GAA microsatellite along the chromosomes of *D*. *antarctica*, and no distinct clusters of the sequence, which could be used for chromosome identification, were detected.

The repetitive DNA sequence originally isolated from rye, pSc119.2, was found in the genomes of many species of the tribe *Triticeae* [35, 76–77]. Also, FISH analysis revealed dispersal distribution of the pSc119.2 probe along the chromosomes of *Avena sativa* [78]. The clone pAs1 (*Afa*-family repeat isolated from *Ae*. *tauschii*) is widely spread in the members of *Hordeinae* and *Triticinae* [34, 74, 79–80], but the presence of these repeats in genomes of the members of the *Aveneae* or *Poeae* has not been studied yet. According to our results, FISH analysis did not reveal distinct hybridization sites of both satellite DNA sequences pAs1 and pSc119.2 on chromosomes of *D*. *antarctica*.

The obtained data suggest that either the repeats related to pSc119.2 or pAs1 had been lost in *D*. *antarctica* genome or they had diverged significantly and, therefore, they can not be detected by FISH. Alternatively, they could appear in the phylogenetic lineages *Hordeinae* and *Triticinae* after their divergence from the lineages *Aveneae* and/or *Poeae*. For a better understanding of the phylogenetic position of the genus *Deschampsia* within the family *Poaceae* as well as clarifying the relationships within the genus, further molecular cytogenetic studies of *Deschampsia* species are required.

## Conclusions

The karyotype variability found in *D*. *antarctica* specimens collected on four islands of the Maritime Antarctic is probably an expression of genome instability induced by environmental stress factors. The revealed intercalary site of telomere repeats confirms indirectly the hypothesis that chromosome fusion might have been the cause of the unusual for cereals chromosome number in this species. The differences in C-banding patterns and in chromosome distribution of rDNA loci as well as homologous highly repeated DNA sequences detected between genomes of *D*. *antarctica* and its related species *D*. *caespitosa* indicate that genome reorganization involving coding and noncoding repeated DNA sequences had occurred during the divergence of these species. The molecular cytogenetic analysis of chromosomes of *D*. *antarctica* performed in this study is important for further comparative cytogenetic studies of *Deschampsia* species and clarifying the relationships within the genus. Also, the obtained data provide a basis for genetic and biotechnological applications and would be useful for breeding cold tolerance in crops.
